# Safflower Yellow B Protects Brain against Cerebral Ischemia Reperfusion Injury through AMPK/NF-kB Pathway

**DOI:** 10.1155/2019/7219740

**Published:** 2019-02-03

**Authors:** Shibin Du, Youliang Deng, Hongjie Yuan, Yanyan Sun

**Affiliations:** ^1^Department of Anesthesiology, Shenzhen University General Hospital, Shenzhen University Clinical Medical Academy, Shenzhen University, Shenzhen 518055, China; ^2^Department of Anesthesiology, Xinqiao Hospital, Third Military Medical University, Chongqing 400037, China; ^3^Department of Pain Medicine, Nantong Hospital of Traditional Chinese Medicine, Nantong 226001, China

## Abstract

Inflammation had showed its important role in the pathogenesis of cerebral ischemia and secondary damage. Safflower yellow B (SYB) had neuroprotective effects against oxidative stress-induced brain injuries, but the mechanisms were still largely unknown to us. In this study, we tried to investigate the anti-inflammation effects of SYB and the possible roles of AMPK/NF-*κ*B signaling pathway on these protective effects. In vivo, brain ischemia/reperfusion (I/R) was induced by transient middle cerebral artery occlusion for 2 h and reperfusion for 20 h. Neurofunctional evaluation, infarction area, and brain water contents were measured. Brain injury markers and inflammatory cytokines levels were measured by ELISA kits. In vitro, cell viability, apoptosis, and LDH leakage were measured after I/R in PC12 cells. The expression and phosphorylation levels of AMPK, NF-*κ*B p65, and P-I*κ*B-*α* in cytoplasm and nuclear were measured by Western blotting. SiRNA experiment was performed to certify the role of AMPK. The results showed SYB reduced infarct size, improved neurological outcomes, and inhibited brain injury after I/R.* In vitro* test, SYB treatment alleviated PC12 cells injury and apoptosis and inhibited the inflammatory cytokines (IL-1, IL-6, TNF-*α*, and COX-2) in a dose-dependent manner. SYB treatment induced AMPK phosphorylation and inhibited NF-*κ*B p65 nuclear translocation both in brain and in PC12 cells. Further studies also showed that the inhibition of NF-*κ*B activity of SYB was through AMPK. In conclusion, SYB protected brain I/R injury through reducing expression of inflammatory cytokines and this effect might be partly due to the inhibition of NF-*κ*B mediated by AMPK.

## 1. Introduction

Around the world, ischemia stroke had been the third leading cause of death and the leading cause of disability [1]. Cerebral ischemia was always caused by reduction or blockage of the blood flow to some regions in brain which is induced by vascular reflow after contraction, percutaneous transluminal coronary angioplasty, organ transplantation, and so on [2]. Clinically, vascular recanalization was always used to restore blood supply; however, this reperfusion might induce further injuries in brain, and this process was called ischemia/reperfusion (I/R) injury [3]. I/R caused multiple insults to cerebral microvasculatures which are accompanied with oxygen free radicals production, mast cell degranulation, and endothelial cell injury [4]. It had been established that inflammatory response plays an important role in the pathogenesis of cerebral I/R injury [5]. Therefore, pharmacological alleviation of inflammatory damage was considered to be one of the most promising solutions for the treatment of stroke.

Nuclear factor-kappa B (NF-*κ*B), a critical transcription factor involved in the inflammatory processes, was activated during I/R which further induced a variety of proinflammatory molecules expression, such as interleukin-1 (IL-1), IL-6, tumor necrosis factor-a (TNF-a), and resulted in neuron death [6]. AMP-activated protein kinase (AMPK) had been recognized as an emergency response enzyme and played important roles at the cellular and whole-organism levels [7]. Some studies had showed that AMPK protected brain from I/R injuries though inhibiting NF-*κ*B mediated inflammatory [8]. Thus, AMPK is currently proposed as a new therapeutic target for I/R-induced inflammation.

*Carthamus tinctorius* L. (safflower) had been frequently used in traditional Chinese medicine for treatment of cerebrovascular and cardiovascular diseases [9–11]. It also exerts other pharmacological effects such as anticoagulant, antioxidant, and calcium antagonist effects. It had been reported that the main chemical constituents in safflower were flavonoids, polysaccharides, lignans, and triterpene alcohols [12]. The extracts from safflower contained yellow and red pigments which included safflower yellow B (SYB), hydroxysafflor yellow A (HSYA), safflower yellow A (SYA), and others [13]. However, which components are responsible for its protective effects were still largely unknown for us. SYB, a natural flavonoid compound, had been used as cardiovascular drugs in traditional Chinese medicine [14]. Some literatures reported that SYB had strong antioxidant effects and protected oxidative stress-induced nerve and hepatocytes cell damage [15, 16]. However, little research on the anti-inflammatory effects of SYB on brain I/R had been undertaken. Thus, in this study, we tried to investigate (1) whether SYB inhibit inflammatory mediated by I/R* in vivo* and* in vitro*; (2) whether the anti-inflammatory effect of SYB was mediated by AMPK/NF-*κ*B signaling pathway.

## 2. Materials and Methods

### 2.1. Materials

SYB (purity >98%, determined by HPLC) was obtained from the Chinese National Institute for the Control of Pharmaceutical and Biological Products (Beijing, China). Compound C was obtained from Toronto Research Chemicals Inc. (Toronto, ON, Canada). Cell culture related reagents including Dulbecco's modified Eagle's medium (DMEM) and fetal bovine serum (FBS) were purchased from HyClone (Logan, UT, USA). Cell counting kit (CCK-8), type I collagenase, and western blot reagents were obtained from Sigma (St. Louis, MO, USA). The kits for the determination of interleukin-6 (IL-6), IL-1, tumor necrosis factor-*α* (TNF-*α*), and Cyclooxygenase 2 (COX-2) were purchased from Nanjing Jiancheng Bioengineering Institute (Nanjing, China). The antibodies against AMPK, P-AMPK, ACC, P-ACC, NF-*κ*B, I-*κ*B, Bax, Bcl-2, cleaved-caspase 3, and *β*-actin were obtained from Santa Cruz Biotechnology, USA. All other chemicals used were of the best available commercial grade.

### 2.2. Rat Ischemia/Reperfusion (I/R) Model

All animal experiments were conducted according to “The Guidance to Experimental Animal Welfare and Ethical Treatment” by The Ministry of Science and Technology of the People's Republic of China (2006) and “Guide for the Care and Use of Laboratory Animals” by the National Research Council of the National Academies (National Academy of Science, 2011).

Rats were randomly divided into 6 groups: sham, model, SYB-L (treated with 2 mg/kg SYB), SYB-M (treated with 4 mg/kg SYB), SYB-H (treated with 8 mg/kg SYB), and NIM (nimodipine-treated group, 0.4 mg/kg). The focal ischemia was induced by middle cerebral artery occlusion (MCAO) and performed as described in detail previously [17]. Briefly, rats were anaesthetized by intraperitoneal injection of chloral hydrate (300 mg/kg). Body temperature and vital signs were monitored. The middle cerebral artery (MCA) was isolated and occluded by a monofilament nylon suture for 2 h. The rats in sham group were underwent the same surgical procedure expect for arterial occlusion. After reperfusion for 20 h, rats were euthanized and blood and brain tissues were collected. The drug was administrated by intraperitoneal injection. In sham and model rats, saline was given by the same administration.

### 2.3. Cerebral Infarction and Neurofunctional Evaluation

After reperfusion for 20 h, the neurological deficit score of rats was evaluated by Longa's method of a five-point scale [17]. The evaluation was performed by a researcher who was blinded to the design: 0 = no neurological deficit; 1 = failure to extend right paw fully; 2 = circling to right; 3 = falling to right; 4 = no spontaneous walking with a depressed level of consciousness.

Postural reflexes after reperfusion were measured as the method reported [18]. Briefly, rats were suspended 100 cm above the ground surface and slowly lowered and then placed on the table and pushed from side to side: 0 = no deficit; 1 = forelimb flexion when rats were suspended by the tail; 2 = decreased resistance to lateral push.

The beam balance test was also used [19]. After reperfusion, rats walked on a narrow beam (width 1.5 cm; length 150 cm) and the behavior was scored as the method reported [20]: 1 = steady posture with paws and balance on the beam; 2 = grasps side of beam or wavering; 3 = hugs the beam and one or two limbs slip off beam; 4 = hugs the beam and three limbs slip off beam; 5 = rat attempts to balance with paws on beam but falls; 6 = rat drapes over beam, then falls; 7 = rat falls off the beam without attempting to balance.

Adhesive removal test was used to measure the sensory stimuli response [21]. A small piece of adhesive tape square (width 2 mm; length 2 mm) was placed on right or left C2 or C3 vibrissae of the rat. The right or left was alternated between each rat and each session, and then the times to contact or remove the adhesive tape were measured, with a limit of 60 s.

Following the neurological evaluation, the brain was collected from 6 rats in each group and kept at -20°C for 40 min. Brains were sliced into five slices of 2 mm thick and then stained in 2% solution of 2,3,5-tri-phenyl tetrazolium chloride (TTC) at 37°C for 10 min. The stained slices were photographed and measured by an image analysis system (JM VIS-TEC, Suzhou, China). The infarct volumes were expressed as the ratio virus the contralateral hemisphere volume.

### 2.4. PC12 Cells Ischemia/Reperfusion (I/R) Model

PC12 cells were cultured by DMEM supplemented with 10% fetal bovine serum at 37°C in 5% CO_2_ and 95% air. The culture medium was changed every other day. To initiate ischemia/reperfusion (I/R) model in vitro, cells were plated into relative culture dishes and cultured for 24 h. After treatment with SYB for another 24 h, the medium was removed and replaced with glucose-free DMEM in a hypoxia incubator (95% N_2_ and 5% CO_2_) for 4 h (ischemia). Following ischemia, the glucose-free DMEM was replaced with the normal culture medium and cells were incubated for another 20 h (reperfusion) under normal culture conditions.

### 2.5. Analysis of Cell Viability

MTT assay was used to determine cell viability. PC12 cells were plated into 96-well plates at a density of 1 x 10^4^ cells/well. Cells were treated by different drugs for 24 h and subjected to I/R. 20 *µ*l MTT solution (5 mg/ml) was added to each well and maintained at 37°C for 4 h. The MTT was removed and 150 *μ*l DMSO was added to each well. The optical density (OD) was determined by a microplate reader (Infinite M200 PRO, Tecan) at 490 nm. The cell survival ratios were showed as a percentage of the control.

### 2.6. Cell Apoptosis

PC12 cells were pretreated with SYB for 24 h before subjected to I/R. The cells were washed by PBS and treated according to manufacturer's instructions for the Annexin V-FITC apoptosis assay kit. The apoptosis rate was analyzed by a flow cytometry (BD Biosciences, San Jose, CA, USA). Cell apoptosis ratio was expressed as percentages of the number of total cells.

### 2.7. LDH Leakage

The cytotoxicity was measured by the LDH leakage assay after different treatments. The culture medium was collected, and cells were scraped in PBS. The cells membranes were broken down to release the total LDH in cells, and then centrifugation was undertaken to clear up the cell samples. The LDH leakage was calculated from the ratio between the LDH levels in culture medium and in cell content.

### 2.8. Cytokine Enzyme-Linked Immunosorbent Assay (ELISA)

Serum or culture supernatants were collected and saved at -20°C until used. ELISA for IL-1, IL-6, TNF-*α*, and COX-2 were used to measure the anti-inflammation activity of SYB. All operations were performed according to manufacturer's protocol.

### 2.9. Protein Extraction and Western Blot Analysis

Total or nuclear proteins extracts of different cell treatment group and brain tissue were lysed by nuclear and cytoplasmic extraction reagent kits. The protein concentrations were determined by a BCA protein measurement kit. Equal amounts of protein samples (30 *µ*g) were separated by 10% sodium dodecyl sulfate-polyacrylamide gel electrophoresis (SDS-PAGE) and electroblotted onto polyvinylidene difluoride (PVDF) membranes. The protein membranes were blocked with 5% non-fat dried milk for 30 min at 37°C, followed by incubation with specific primary antibodies (AMPK, P-AMPK, NF-*κ*B, P-I*κ*B-*α*, Bax, Bcl-2, cleaved-caspase 3, and *β*-actin) overnight at 4°C. Then, the membranes were further incubated with relative secondary antibody for 1 h at 37°C. Bands were detected using enhanced chemiluminescence light-based detection kits (Millipore, Billerica, MA, uSA). *β*-Actin was used as a loading control in cytoplasm. Lamin B was used as a loading control in nuclear.

### 2.10. Statistical Analysis

Data are presented as mean ± SD. One-way analysis of variance (ANOVA) was used followed by Tukey's multiple comparison tests using Graphpad prism 5.0. P-values of <0.05 and <0.01 were considered statistically significant.

## 3. Results

### 3.1. SYB Protected Brain from I/R-Induced Injuries

**Figure 1:**
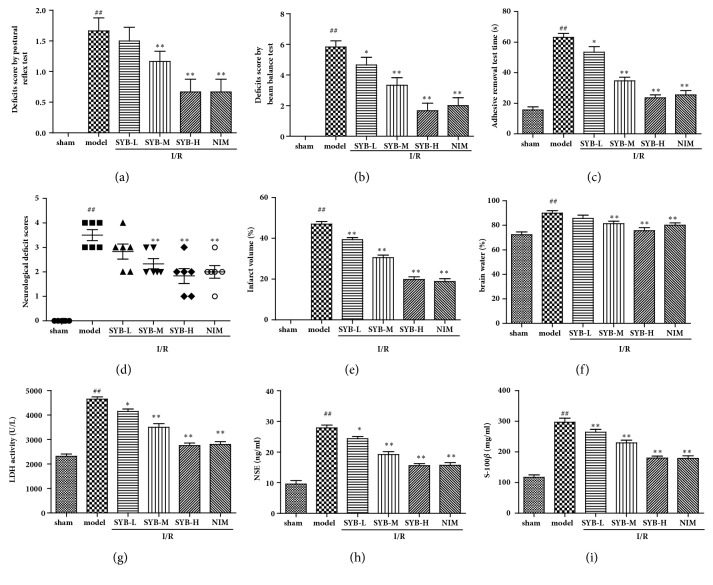
Neuroprotective effect of SYB on I/R-induced brain injuries. Animals were given SYB (2, 4, and 8 mg/kg) by intraperitoneal injection before reperfusion for 20 h (a). The postural reflex test results (b). The beam balance test results (c). Adhesive removal test results (d). The neurological deficit score of rats was evaluated according to Longa's method (e). Infarction area was measured by TTC staining (f). Brain water content was measured as MOTHED shown. After reperfusion, the blood was collected and the levels of LDH (g), NSE (h), and S-100*β* (i) were measured by relative kits as the protocol directed. Data were presented as mean ± SD. ^##^P<0.01 versus sham group; ^*∗*^P < 0.05; ^*∗∗*^P < 0.01 versus sham group.

To evaluate the effects of SYB on the balance and sensory activity, postural reflexes, adhesive removal test, and beam balance test were performed after stroke. As the results showed, rats got high scores on reflex (Figure 1(a)), beam balance (Figure 1(b)), and sensory tests (Figure 1(c)), indicating that the balance and sensory activity were injured by I/R treatment. As compared with the model group, the scores were significantly decreased by SYB treatment (P<0.01). The neurological deficit scores were also measured. After reperfusion, neurological deficit scores in I/R rats were significantly higher than those in sham group (P<0.01), indicating that I/R effectively caused neurological dysfunction in rats (Figure 1(d)). As compared with the model group, neurological deficit scores were significantly decreased in SYB and NIM treatment groups (P<0.01). Infarct volume was measured by TTC after reperfusion. No infarction was observed in sham group. In model group, the infarct volume was significantly increased by I/R treatment (P<0.01). Treatments with SYB at all doses significantly reduced the infarct volume compared with the model group and showed dose-dependent manner (Figure 1(e)). Following I/R, there was a significant reduction in brain water content in the SYB treatment groups compared with model group (Figure 1(f)). LDH, S-100*β*, and NSE levels in brain were considered as brain injury markers. Compared with the sham group, I/R significantly increased the levels of LDH, S-100*β*, and NSE in brain, and SYB treatments significantly decreased their levels compared with model group (Figures 1(g), 1(h), and 1(i)).

### 3.2. SYB Inhibited I/R-Induced Inflammatory Reaction in Brain

**Figure 2:**
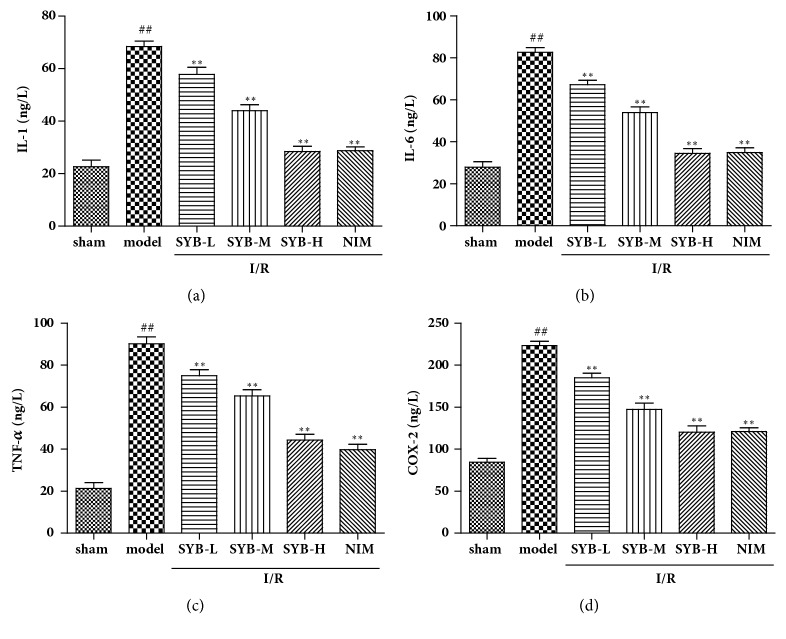
Effects of SYB on the inflammatory factors levels in rats subjected to brain I/R. After reperfusion, the blood of brain tissues was collected and the levels of IL-1, IL-6, TNF-*α*, and COX-2 were measured by relative kits as the protocol directed. Data were presented as mean ± SD. ^##^P<0.01 versus sham group; ^*∗*^P < 0.05; ^*∗∗*^P < 0.01 versus sham group.

To determine whether SYB had some effects on the inflammatory reaction in brain after I/R, inflammatory factors including IL-1, IL-6, TNF-*α*, and COX-2 were measured by ELISA. As shown in Figure 2, levels of IL-1, IL-6, TNF-*α*, and COX-2 in serum were significantly increased in model group, which compared with sham group (P<0.01). After treatment with SYB or NIM, serum levels of IL-1, IL-6, TNF-*α*, and COX-2 were significantly inhibited compared with model group (P<0.01). These results demonstrated that I/R induced inflammatory reaction in brain, and SYB treatments had protective effects on I/R-induced inflammatory reaction.

### 3.3. SYB Induced AMPK Phosphorylation and Inhibited NF-*κ*B Expression in Brain

**Figure 3:**
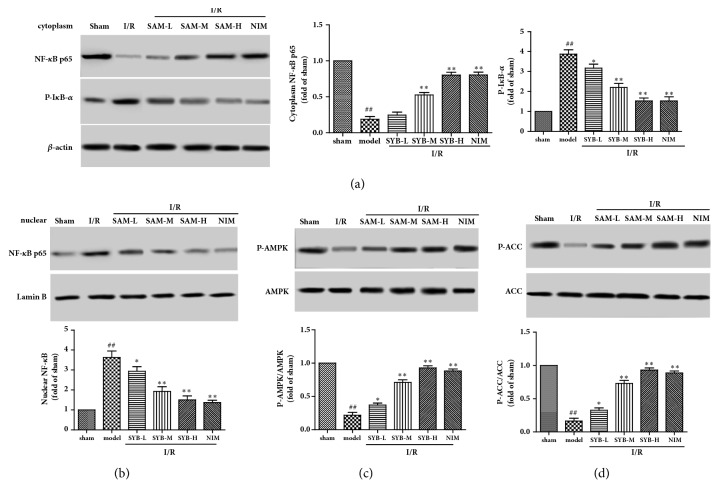
Effects of SYB on the expression of NF-*κ*B in the nuclear and cytoplasm and the phosphorylation levels of AMPK and ACC. After reperfusion, the brain tissues in every groups were collected and the proteins in nuclear and cytoplasm were lysed by nuclear and cytoplasmic extraction reagent kits (a). Effects of SYB on the expression levels of NF-*κ*B p65 and P-I*κ*B-*α* in cytoplasm (b). Effects of SYB on the expression levels of NF-*κ*B p65 in nuclear (c). Effects of SYB on the phosphorylation levels of AMPK (d). Effects of SYB on the phosphorylation levels of ACC. Data were presented as mean ± SD. ^##^P<0.01 versus sham group; ^*∗*^P < 0.05; ^*∗∗*^P < 0.01 versus sham group.

To determine the effect of SYB on NF-*κ*B p65 expression, levels of NF-*κ*B p65 were measured in cytoplasm and nuclear. As the results showed in Figure 3(a), the expression levels of NF-*κ*B p65 were decreased in cytoplasm and increased significantly in nuclear, compared with sham group (P<0.01). The phosphorylation levels of I*κ*B-*α* were increased significantly in model group, compared with sham group (P<0.01). In SYB treatment group, the expression levels of NF-*κ*B p65 were increased in cytoplasm and decreased in nuclear compared with model group (P<0.01). The phosphorylation levels of I*κ*B-*α* were also inhibited by SYB treatments.

Next, the phosphorylation levels of AMPK and its downstream ACC were measured by Western blotting. As the results showed in Figure 3(b), I/R induced significant reduction of AMPK phosphorylation levels and also ACC phosphorylation levels in model group compared in sham group (P<0.01). However, in SYB treatment group, the phosphorylation levels of AMPK and ACC were significantly increased compared with model group (P<0.01). These results suggested that SYB could induce AMPK phosphorylation and inhibited NF-*κ*B expression in brain subjected to I/R injury.

### 3.4. SYB Protected PC12 Cells from I/R-Induced Cell Apoptosis

**Figure 4:**
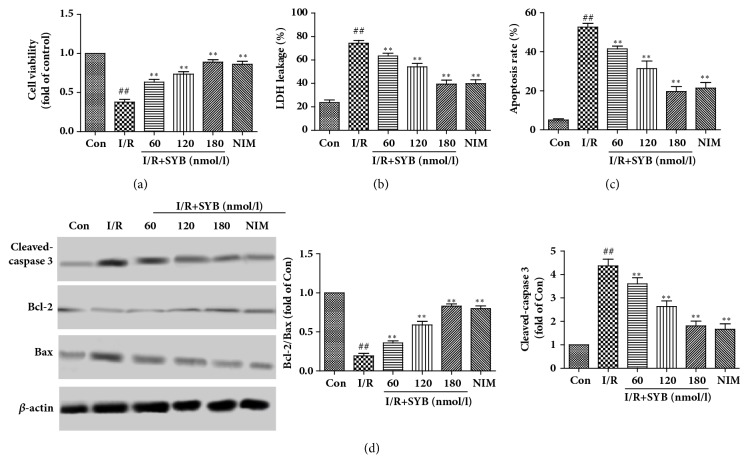
SYB pretreatment protected PC12 cells from I/R-induced cytotoxicity and apoptosis (a). Protective effects of SYB on I/R-induced cytotoxicity in PC12 cells. PC12 cells viability was assessed by measuring the MTT reduction. Results were shown as fold of control (b). Effects of SYB on the LDH leakage induced by I/R (c). Effects of SYB on PC12 cells apoptosis induced by I/R. PC12 Cells were subjected to I/R with or without SYB pretreatment, then double stained with Annexin V/propidium iodide (PI) (d). The expression levels of cleaved-caspase 3, Bax, and Bcl-2 were measured by Western blotting. Data were presented as mean ± SD. ^##^P<0.01 versus control group; ^*∗∗*^P < 0.01 versus I/R group.

To elucidate the protective effects of SYB against I/R-induced cell damage, PC12 cells were pretreated with SYB (60, 120, and 180 nmol/l) for 24 h and then subjected to I/R for another 24 h. Concentrations of SYB used in this study did not induce any adverse effects (data not shown). The protective effects of SYB on I/R induced cell injuries were determined by MTT assay and LDH leakage assay. As the results showed in Figure 4(a), cell viability was significantly decreased by I/R treatment, and pretreatment with SYB for 24 h increased the cell viability in a dose-dependent manner. Similarly, the LDH leakage assay revealed that I/R induced LDH leakage from the cells, and SYB inhibited LDH leakage in a dose-dependent manner (Figure 4(b)), suggesting that SYB enabled PC12 cells to maintain cell integrity.

To determine whether SYB had protective effects against apoptosis, Annexin V-FITC/PI double staining and western blotting were used. As the results of flow cytometric detection showed, compared with control group, I/R induced a significant increase in the apoptosis of PC12 cells, and SYB pretreatment significantly decreased the apoptosis rate (Figure 4(c)). The results of western blotting showed that I/R obviously reduced Bcl-2 expression and increased Bax and cleaved-caspase 3 expression compared with control group (Figure 4(d)). Compared with model group, treatment of PC12 with SYB increased Bcl-2 expression and decreased Bax and cleaved-caspase 3 expression (P<0.01).

### 3.5. SYB Inhibited I/R-Induced Inflammatory Factors Expression in PC12 Cells

**Figure 5:**
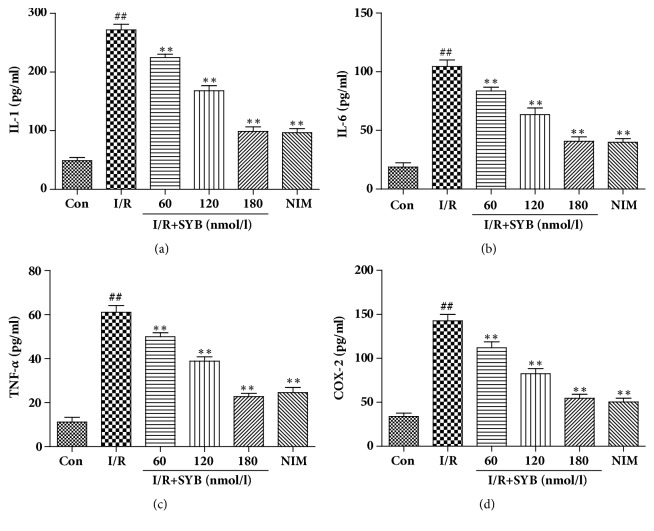
SYB pretreatment protected PC12 cells from I/R-induced inflammatory response. PC12 cells were pretreated by SYB for 24 h and then subjected to I/R. The cell culture medium was collected and levels of IL-1, IL-6, TNF-*α*, and COX-2 were measured by ELISA kits. Data were presented as mean ± SD. ^##^P<0.01 versus control group; ^*∗∗*^P < 0.01 versus I/R group.

To study the mechanisms through which SYB performs its protective effects, levels of inflammatory factors were determined. The PC12 cells were pretreated with SYB for 24 h and subjected to I/R for another 24 h, and the levels of IL-1, IL-6, TNF-*α*, and COX-2 were measured by ELISA kits. The results showed that I/R induced rapid and significant increase of IL-1, IL-6, TNF-*α*, and COX-2 levels in PC12 cells (Figure 5). Pretreatment of the cells with SYB for 24 h significantly decreased the levels of IL-1, IL-6, TNF-*α*, and COX-2 compared with the I/R group (P<0.01). These results suggested that SYB had protective effects against I/R-induced inflammatory response in PC12 cells.

### 3.6. The Effects of SYB on the Expression of AMPK and NF-*κ*B in PC12 Cells

**Figure 6:**
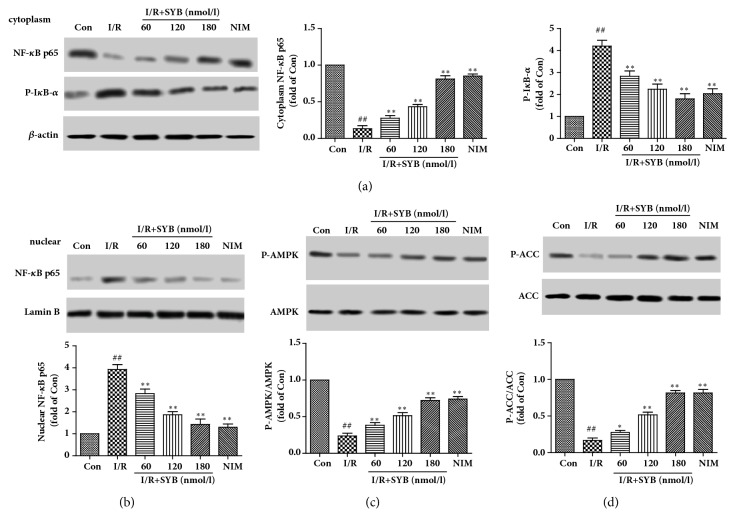
Effects of SYB on the AMPK/ NF-*κ*B pathway in PC12 cells. PC12 were pretreated with SYB and subjected to I/R for 24 h; then the proteins in nuclear and cytoplasm were lysed by nuclear and cytoplasmic extraction reagent kits (a). Effects of SYB on the expression levels of NF-*κ*B p65 and P-I*κ*B-*α* in cytoplasm of PC12 cells (b). Effects of SYB on the expression levels of NF-*κ*B p65 in nuclear of PC12 cells (c). Effects of SYB on the phosphorylation levels of AMPK in PC12 cells (d). Effects of SYB on the phosphorylation levels of ACC in PC12 cells. Data were presented as mean ± SD. ^##^P<0.01 versus control group; ^*∗*^P < 0.05; ^*∗∗*^P < 0.01 versus I/R group.

NF-*κ*B signaling pathway was an important inflammatory regulator responsible for I/R. To clarify the mechanism of SYB in inhibiting inflammation, the activity of NF-*κ*B was measured by western blotting. As shown in Figure 6(a), there was a low basal level of NF-*κ*B p65 in the nuclear of normal cells. I/R induced significant increasing of NF-*κ*B p65 in the nuclear together with an increased phosphorylation levels of I-*κ*B, suggesting I/R induced a translocation of NF-*κ*B p65 from the cytoplasm to the nucleus. In addition, SYB inhibited the phosphorylation of I-*κ*B and nuclear translocation of NF-*κ*B p65 in a dose-dependent manner.

AMPK had showed its protective effects on I/R related injuries. The phosphorylation of AMPK was also determined by Western blotting. As shown in Figure 6(b), the phosphorylation levels of AMPK were significantly lower in I/R group than those in control group (P<0.01), while the phosphorylation levels of AMPK were increased by SYB pretreatment in a dose-dependent manner as well as its downstream ACC. These results suggested that SYB induced the phosphorylation of AMPK and activated the downstream pathway.

### 3.7. SYB Inhibited the Nuclear Translocation of NF-*κ*B through AMPK

**Figure 7:**
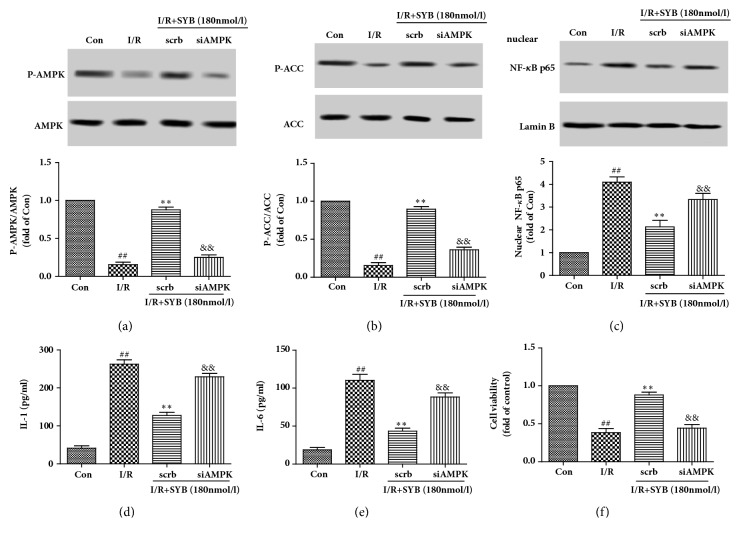
The relationships between AMPK and NF-*κ*B in PC12 cells subjected to I/R. PC12 cells were transfected with siRNAs directed against AMPK (siAMPK) or nontargeting control sinRNA (scrb), continuously incubated with SYB for 24 h, and then subjected to I/R. The proteins in nuclear and cytoplasm were lysed by nuclear and cytoplasmic extraction reagent kits (a). Effects of SYB on the phosphorylation levels of AMPK in PC12 cells after siAMPK translation (b). Effects of SYB on the phosphorylation levels of ACC in PC12 cells after siAMPK translation (c). Effects of SYB on the expression levels of NF-*κ*B p65 in nuclear of PC12 cells after siAMPK translation. siAMPK abolished the effects of SYB on the levels of IL-1 (d), IL-6 (e), and cell viability (f). Data were presented as mean ± SD. ^##^P<0.01 versus control group; ^*∗∗*^P < 0.01 versus I/R group; ^&&^P<0.01 versus SYB treatment group.

In order to further determine the role of AMPK on the anti-inflammatory effects of SYB, the AMPK specific siRNA, siAMPK were used to block the activation of AMPK. As the results showed in Figures 7(a) and 7(b), siAMPK inhibited the SYB-induced phosphorylation of AMPK and ACC in the PC12 cells subjected to I/R. Moreover, the inhibition of AMPK markedly reduced the capacity of SYB to decrease NF-*κ*B p65 nuclear translocation (Figure 7(c)), as well as the IL-1 and IL-6 levels (Figures 7(d) and 7(e)). Consistently, the inhibition of AMPK by siAMPK also eliminated the SYB induced cytoprotective effects against I/R induced cell viability reduction (Figure 7(f)). These results suggested that SYB inhibited the NF-*κ*B mediated inflammatory response by activating the AMPK pathway.

## 4. Discussion

Cerebral ischemia was one of the most common clinical circulatory arrests and caused neuronal damage in some certain vulnerable brain areas like hippocampal CA1 region [22]. Several mechanisms were included in cerebral ischemia induced injury, including calcium overload, inflammatory response, free radical injury, and some others [23]. Among these, inflammatory response was considered to be the leading cause of cerebral ischemia [24]. Cerebral ischemia induced inflammatory response and reperfusion aggravated the inflammation response causing secondary brain damage [25]. Proinflammatory responses were found within minutes after the onset of cerebral ischemia and lead to tissue damage, improper cellular repair, and dysfunction [26]. So inhibition of the inflammatory response in ischemia regions at the beginning of ischemia might provide effective treatment.

Safflower was always used for the treatment of coronary heart disease, hypertension, and cerebrovascular disease [27]. Previous studies considered that HSYA was the main active substance; however, more and more researchers found that other water-soluble compounds including SYB and SYA were responsible for its therapeutic effects [13, 14]. Safflower injection had been approved by the State Food and Drug Administration of China for the treatment of cerebral ischemia in 2000. Clinical studies had showed that safflower injection had good curative effects and minor side effects [28]. There were some literatures reported that HSYA had anti-inflammatory effects of and protected brain from I/R injury [29, 30]. However, to the best of our knowledge, there are no studies available to date on the effects of SYB on I/R-induced inflammatory response in brain. Thus, this study was designed to examine the inhibitory effects of SYB on I/R-induced inflammatory response in vivo and in vitro.

In this study, MCAO and reperfusion in rat were used to mimic some features of human brain pathology. The results of neurological deficit scores and infarct volume showed that I/R model was successful. Treatment with SYB significantly decreased the neurological deficit scores and infarct volume and showed dose-dependent manner. Serum neuron specific enolase (NSE) and S100 calcium binding protein B (S-100B), two biochemical markers of brain injury, were always used clinically to evaluate the degree of brain injury [31]. In this study, we found that I/R increased the levels of NSE and S-100B, and SYB decreased their levels in a dose-dependent manner. These results suggested that SYB had protective effects against I/R-induced brain injuries.

Proinflammatory cytokines were upregulated in the brain after some pathological stimulus including cerebral ischemia. They were secreted by immune cells and also produced by brain cells, like glia cells and neurons [32, 33]. The most important inflammation related cytokines in brain during ischemia injury were IL-1, IL-6, COX-2, and TNF-*α* [34]. Among these, IL-1 and TNF-*α* could exacerbate the degree of brain injury [35]. In the current study, we found that IL-1, IL-6, COX-2, and TNF-*α* were increased significantly after I/R operation. SYB treatment inhibited the elevation of IL-1, IL-6, COX-2, and TNF-*α* in brain and PC12 cells. These results suggested that SYB inhibited the inflammation induced by I/R in vivo and in vitro.

In eukaryotic cells, NF-*κ*B was an important nuclear transcription factor which regulates the expression of many cytokines, including proinflammatory cytokines [36]. In various diseases, NF-*κ*B had been found to be a promising target in inhibiting inflammatory response. Accumulating evidences showed that elevated NF-*κ*B contributes to brain injury induced by ischemia [37]. Under normal condition, NF-*κ*B was associated with its inhibitory protein I*κ*B and sequestered in the cytoplasm. Upon activation, I*κ*B kinases (IKKs) were activated and led to the phosphorylation of I*κ*B which further induced the proteasome-mediated degradation; thus NF-*κ*B is transported into nucleus from cytoplasm [38]. The nuclear NF-*κ*B bound with its binding sequence to activate the relevant promoters and induced the expression of inflammatory cytokines, including IL-1, IL-6, COX-2, and TNF-*α* [39]. These facts suggested that NF-*κ*B played an important role in regulating inflammation, and the inhibition of NF-*κ*B was protective against neuroinflammation and neurodegeneration. In this study, I/R induced the phosphorylation of I*κ*B and nuclear translation of NF-*κ*B p65 in brain and PC12 cells. However, SYB treatments significantly decreased the nuclear translation of NF-*κ*B p65, together with the reduction of I*κ*B phosphorylation. These results suggested that the anti-inflammation effects of SYB might be through inhibiting the NF-*κ*B pathway.

AMPK had been considered as a detector of cellular homeostasis and also modulated oxidative stress and inflammation [40]. It regulated several signal translocation pathways to affect the cell death and survival. AMPK could also mediate several signaling cascades to inhibit the inflammation [41]. The results of Western blotting showed that SYB treatment significantly increased the phosphorylation of AMPK and also its downstream ACC, suggesting SYB could activate the AMPK pathway. Amassing research supported that AMPK negatively regulates NF-*κ*B, and the reduced AMPK led to an increase of NF-*κ*B signaling activities in several cell lines [42, 43]. Therefore, we investigated whether that AMPK pathway contributes to the protective effects of SYB. To further study the relationship between AMPK and NF-*κ*B during SYB treatment, compound C and siAMPK were used. The results showed that inhibition of AMPK markedly reduced the capacity of SYB to decrease NF-*κ*B p65 nuclear translocation and increased the expression level of IL-1 and IL-6. Further analysis also indicated that siAMPK abolished the cytoprotective effects of SYB against I/R injury. These results suggested that AMPK/NF-*κ*B was involved in the cytoprotective effects of SYB.

In conclusion, our results strongly suggested that SYB treatment protected cerebral cell from I/R induced inflammation through a mechanism that SYB activated AMPK and negatively regulated NF-*κ*B mediated inflammatory response. These results provided some scientific evidences for the cerebral protection effects of SYB and suggested it might be useful in the treatment of various brain diseases associated with inflammation.

## Data Availability

The data used to support the findings of this study are available from the corresponding author upon request.
